# Dynamic colour change and the confusion effect against predation

**DOI:** 10.1038/s41598-018-36541-7

**Published:** 2019-01-22

**Authors:** Gopal Murali, Kajal Kumari, Ullasa Kodandaramaiah

**Affiliations:** IISER-TVM Centre for Research and Education in Ecology and Evolution (ICREEE), School of Biology, Indian Institute of Science Education and Research Thiruvananthapuram, Maruthamala PO, Vithura, Thiruvananthapuram 695 551 India

## Abstract

The confusion effect - the decreased attack-to-kill ratio of a predator with increase in prey group size - is thought to be one of the main reasons for the evolution of group living in animals. Despite much interest, the influence of prey coloration on the confusion effect is not well understood. We hypothesized that dynamic colour change in motion (due to interference coloration or flash marks), seen widely in many group living animals, enhances the confusion effect. Utilizing a virtual tracking task with humans, we found targets that dynamically changed colour during motion were more difficult to track than targets with background matching patterns, and this effect was stronger at larger group sizes. The current study thus provides the first empirical evidence for the idea that dynamic colour change can benefit animals in a group and may explain the widespread occurrence of dynamic colorations in group-living animals.

## Introduction

Group living is a widespread behavior which has evolved independently in many animals (e.g.^1,2^) and predator avoidance is considered as one of the primary selective agents driving the evolution of group living in animals^3^. Although prey aggregations increase the risk of detection by predators^4,5^, there is ample evidence in a wide variety of taxa suggesting that group-living individuals often have higher survival than solitary individuals (e.g. birds^6,7^, mammals^8^, turtles^9^ and insects^10,11^). This advantage of increased survival with an increase in group size is due to multiple reasons, including increased vigilance^12^, dilution of risk^13^, predator mobbing^14^ and the confusion effect^15,16^.

The confusion effect is a phenomenon where the ability of predators to single out and track an individual prey decreases when the prey move in a group, thereby reducing the predator attack-to-kill ratio^15–18^. This has been well studied both in natural and virtual settings^15–19^ and may explain several stunning examples of grouping behavior such as shoaling of fishes (e.g.^20^) and formation of murmuration patterns in bird flocks (e.g.^7^). The confusion effect occurs as a result of limited information processing ability of predators - processing spatial information of multiple targets declines when prey aggregate^18,21^. A variety of predatory taxa have been documented to be affected by this effect (for a review see^15^), including humans^22,23^.

On the other hand, predators have also shown to attenuate this effect, for instance, by attacking targets at the edge of the group^24^ or by attacking phenotypically odd individuals within the prey group - “the oddity effect”^25^. Therefore, it is believed that for confusion effect to be effective, selection might favor phenotypic similarity of individuals within the prey group^18,23^. This is supported in a recent study where Mediterranean killifish (*Aphanius fasciatus*), when given a choice, preferred more homogeneous groups (for colour patterning) over less homogeneous ones^26^. However, the influence of prey coloration *per se* on the confusion effect, and ultimately group-living, remains largely unexplored (but see^27–29^).

**Figure 1:**
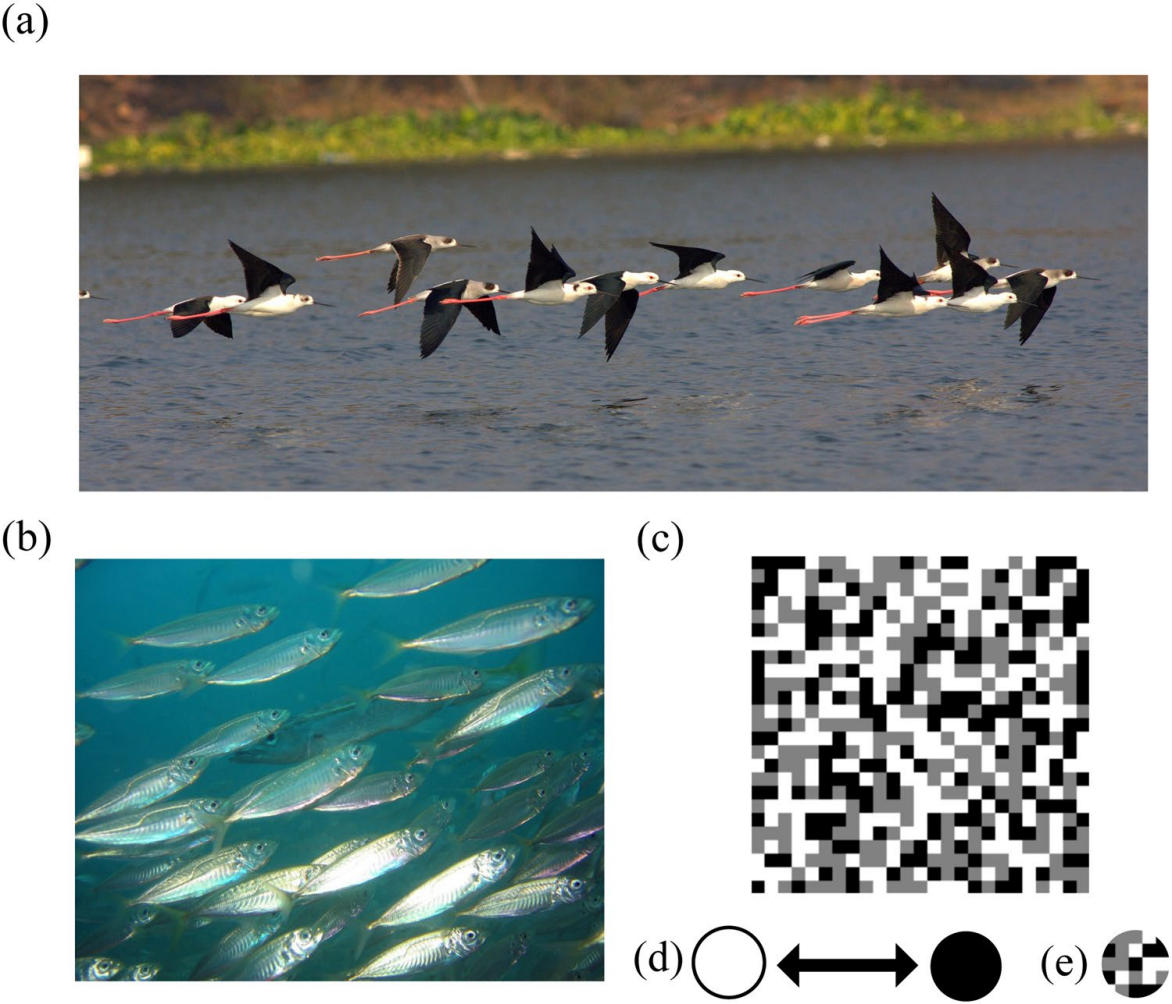
Examples of group living animals with putative dynamic colorations (**a**) Black-winged stilt *Himantopus himantopus* (photo credit: Wikimedia Commons: CC-BY-SA-4.0 (commons.wikimedia.org/wiki/File:Black-winged_stilts_in_flight.jpg) - Rushil Fernandes) (**b**) Jack Mackerel *Trachurus declivis* (photo credit: Wikimedia Commons: CC-BY-SA-2.0 (commons.wikimedia.org/wiki/File:Trachurus_declivis.jpg) - Richard Ling). An exemplar background (**c**), colour dynamic stimulus which switched between black and white over time (**d**) and an exemplar background matching stimulus (**e**) used in the experiment (video of the stimulus presentation can be found online as supplementary to the article).

Many animals such as birds and fishes, which are often found in groups, have colorations that typically change during motion (Fig. 1a,b). This dynamic colour change can either occur due to differential colorations in different body parts for e.g. as in dorso-ventral regions of bird wings (termed flash colorations)^30^ or because of iridescence where the perceived brightness or colour changes based on the illumination angle^31,32^. Previous studies have proposed a range of functions in both intra and interspecific signaling, including sexual and social roles^30–34^. Alternatively, it is suggested that dynamic change of colours can work against predation by hampering prey recognition^35^, by startling predators^36^ or by preventing predators from identifying the final resting location when the prey ceases its movement^37,38^. Recent studies have further shown that dynamic change of colours in motion (due to interference coloration or because of dorso-ventral contrast) in individual prey can reduce predation^39,40^ and that dynamic colour change works by hindering accurate estimation of a prey’s location.

As the spatial targeting error increases with increase in prey group size^18,21^ or density^41,42^, it is likely that colorations that interfere with estimation of the prey’s location during motion further enhance the confusion effect in predators. Therefore, dynamic change of colours is expected to be beneficial against predation by increasing the confusion effect in group living animals (Fig. 1a,b). For instance, Denton^43^ wrote “*Sometimes when a shoal of fish is disturbed by an attacking predator bright flashes can be seen as the fish of the shoal twist and turn and it may be that such flashes distract the predator*, *and shoals of small juvenile fish swimming near the surface may look like ‘rivers’ of silver flashes*.” Further, a comparative study by Brooke^30^ has found the evolution of ‘flash’ marks in shorebirds (e.g., Fig. 1a) to be associated with grouping behavior, indicating that flash coloration might be beneficial in prey aggregations. He suggested that one of the benefits of flash colorations is to enhance the confusion effect - “*This potential benefit of flocking*, *the confusion effect*, *could be enhanced by flash marks if they further distract the predator*. *(…) The confusion effect could also be increased by the habit of some flocking waders of flipping from one side to the other while in flight*, *thereby showing alternately their darker backs and paler bellies*.*”*. However, until now, whether dynamic colour change can reduce predation by increasing the confusion effect has not been experimentally tested.

Here we test the idea that the confusion effect is stronger when targets change colour dynamically than when they do not change colour. We investigated this using an experimental approach from previous studies^22,23,27–29,41^, where human participants were asked to track a single moving target in a group of distractors. If dynamic colour change distracts predators, thereby increasing the confusion effect, we predict that stimuli with dynamic colorations should be more difficult to track compared to conventional background matching patterns. Further, we predict the effect of the dynamic colour change on the confusion effect to increase with an increase in prey group size.

## Results

**Figure 2:**
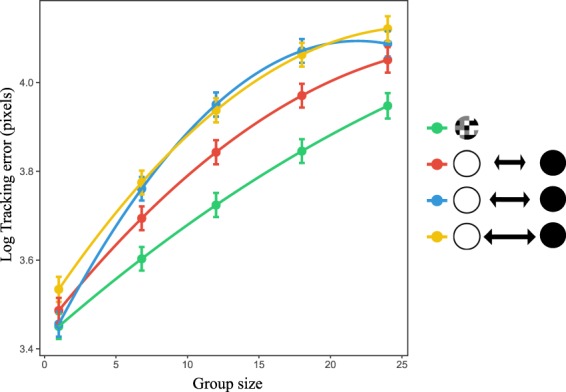
Estimated mean and 95% confidence intervals of log transformed tracking error (in pixels) from the Linear Mixed Effects model. green - background matching stimulus; colour dynamic stimuli with different colour change frequency (red: 5 Hz; blue: 10 Hz; yellow: 15 Hz). For colour dynamic stimuli, the frequency of colour change is represented by the arrow size.

All the main effects included in the Linear Mixed Effects model - stimuli type (χ^2^ = 1690.60, d.f. = 3, *P* < 0.0001) and group size (χ^2^ = 16355.12, d.f. = 2, *P* < 0.0001) - as well as the interaction between the two (χ^2^ = 341.06, d.f. = 6, *P* < 0.0001) were significant. Overall, tracking error increased significantly (t = 55.804, *P* < 0.0001; Fig. 2) with increase in group size (on average 96.84% increase for 1 vs 24). When compared to the background matching stimulus, the tracking error was significantly higher for the colour dynamic stimuli at all colour change frequencies (5 Hz: t = 20.839, *P* < 0.0001; 10 Hz: t = 32.867, *P* < 0.0001; 15 Hz: t = 37.809, *P* < 0.0001; Fig. 2). More importantly, the difference in tracking error between the background matching stimulus and the colour dynamic stimuli increased significantly with increase in group size (5 Hz: t = 5.395, *P* =  < 0.0001; 10 Hz: t = 10.724, *P* < 0.0001; 15 Hz: t = 7.712; *P* < 0.0001). Identical results were obtained when all stimuli changed colour synchronously (Supplementary Section A). In the comparison between the background matching, the colour dynamic and the unicoloured grey stimuli, the trackability of the unicoloured grey stimulus was intermediate to the other two at all group sizes (Supplementary Section B).

## Discussion

The results strongly suggest that visual tracking of a single target was more difficult when the object colour changed dynamically than when the object matched the background, providing additional empirical support for the idea that dynamic change of colour during motion can be an effective antipredator strategy^39,40^. More importantly, the relationship between group size and tracking error was steeper for all the colour dynamic stimuli compared to background matching stimulus. Therefore, the study provides the first clear evidence that dynamic colour change can enhance the confusion effect, likely benefiting prey aggregations with such colorations. The results thus may explain why group living birds and fishes often have flash marks, and support the speculations based on observations made by Denton and Brooke^30,43^.

Moreover, the results also support the previous findings that background matching may not be an effective antipredator strategy during prey motion^44,45^. Although all unicoloured prey (colour dynamic and the grey ones), were more difficult to track compared to the patterned background matching stimulus (Fig. 2, Supplementary Fig. S2), the colour dynamic stimuli was more difficult to track than the unicoloured grey (Supplementary Fig. S2), indicating that the change of colour *per se* increases the confusion effect. Furthermore, background matching stimulus was easier to track than the colour dynamic stimuli irrespective of colour change synchronization (Supplementary Section A; Fig. 2). This is important because in addition to the environmental characteristics, within group similarity is also expected to positively affect the confusion effect^26,28^. Therefore, in the current study, it is possible that for the asynchronous colour change case (Fig. 2), at some time point, certain individual targets within the group did not match the colour of the objects surrounding them, thus creating the “oddity effect”. However, the fact that we found dynamic colour change to be effective irrespective of whether the colour change of the target was “in phase” (synchronous colour change - Supplementary Section A) or “out of phase” (asynchronous colour change - Fig. 2) with other objects within the group suggests that phenotypic homogeneity (i.e. synchronous colour change) in colour change with other objects is not essential for dynamic coloration to be effective. These findings are opposite to what was observed previously for motion dazzle patterns^28^, where the authors find dazzle patterns to have higher confusion effect only when objects in the group have same speed when compared to a group with dazzle patterns having variable speed.

The reason why targets with dynamic coloration are difficult to track could be that the dynamic change of colours might hinder accurate estimation of the target’s position, as hypothesised in other studies^37–40^. Mechanistically, this is similar to a well-known visual illusion termed the flash-lag effect^46–48^, where the position of a flashing target is misperceived when presented along with a non-flashing one^46,47^. This visual illusion is thought to occur due to the inherent delay in neural processing^49^, which illustrates the constraints in estimating the instantaneous position of moving object^40,49^. Therefore, the fact that the tracking was most difficult at high colour change frequency could be because the timing of colour change might better matches the time taken for neural processing than at lower frequencies^50,51^, thus preventing participants from accurately predicting the target position. Further, it is shown that dynamic striped patterns moving within the target affect perceived speed of the moving target^52^. Hence, it is also possible that dynamic change of colour, in addition to hindering accurate estimation of the target’s position^40^, influences perceived speed or direction, which awaits further investigation. Together, the results support the general idea that prey strategies (in the current case colorations) or environmental conditions (for e.g.^53^) that interfere with estimation of prey position enhance the confusion effect. This is also supported by another study^27^, where motion dazzle colorations, which are hypothesised to hinder the estimation of prey speed or direction of movement during the motion, enhanced the confusion effect in groups.

Flicker fusion is a psychophysical phenomenon where, for instance, irregular flashing light with frequency above a certain threshold (critical flicker fusion frequency) is perceived by a visual observer to be continuously lit^54^. Therefore, another possibility is that the colour dynamic stimuli, which switched between white and black at high frequency of colour change, may have been perceived as grey because of flicker fusion^55^, and hence were difficult to track. However, this does not apply to the current results because the frequency of colour change was lower than the critical flicker fusion frequency reported for humans^56^. Further, we also found the dynamic stimuli were more difficult to track than the unicolored average grey stimulus (Supplementary Section B), indicating that the flicker fusion does not explain our results.

Dynamic coloration typically involves combinations of colours that contrast with each other and with the background, and this contrast may make them more visible to predators. Further, prey may attract predator attention because of dynamic change of colours *per se*. Despite these potential costs, dynamic colorations may be a widespread but underappreciated antipredator strategy^35,38–40^. It has been suggested that dynamic colorations in group living animals may help individuals to coordinate escape when there is predation threat^30,31^ or to attract conspecific individuals to resources^33^. While the current results suggest that dynamic coloration can have a beneficial role by increasing the confusion effect, whether this works in synergy with such alternative functions^30,33,34,43^ needs to be studied.

## Materials and Methods

A target tracking task was created in MATLAB R2017a (Mathworks, Natick, MA, U.S.A.) using the Psychophysics Toolbox^57^ as in previous studies^22,27–29,41^. Participants (n = 40) viewed moving circular objects (in sets of varying numbers, i.e. group sizes) of 0.7 cm diameter (0.61°) each, from a distance of ca. 65 cm on a gamma corrected DELL S2240T 21.5-inch touch monitor (40.53° × 23.46°) with a 60 Hz refresh rate. The circular objects moved at a speed of 200 pixels/s (113.95 visual degrees/s) within the central region of the screen (300 × 300 pixels) and bounced off at an angle of π/4 radians on touching the edge. The direction of movement of each circle was changed every video frame and can be defined as a correlated random walk (See Supplementary video and^27–29^). This was achieved by replacing the angle of movement every frame with a randomly drawn value from a circular Gaussian distribution with mean as the angle from the previous frame and the standard deviation as π/12 radians (chosen from pilot experiments).

The background used in the experiments (Fig. 1c) was a heterogeneous pixelated pattern generated in MATLAB following Hogan and colleagues^27^. The absolute grey value of each element (8 × 8 pixels) was 0, 128 or 255 in all three colour channels with the probability of occurrence in the background being 1/3. The experiments had four types of stimuli. One had patterns sampled from the background and therefore matched the background perfectly when stationary (Fig. 1e). Background matching stimulus was colour static, i.e., its colour remained constant, while the remaining three were colour dynamic (Fig. 1d), i.e., they switched between black (R-0, G-0, B-0; average luminance = 5.94 cd/m^2^) and white (R-255, G-255, B-255; average luminance = 139.88 cd/m^2^) over time as a function of a square wave mimicking colour change in some animals with dyanmic colorations (Fig. 1a,b). The colour dynamic stimuli had a colour change frequency of 5 Hz, 10 Hz or 15 Hz to check whether the results were consistent across different frequencies. Because it is unlikely that all members of a prey group change colour synchronously (see for instance Fig. 1a,b), the phase of the square wave defining the colour change for each circle was offset from each other by an amount defined by the ratio of the wavelength to group size (for presentations with group size >1). Results for a colour dynamic target with synchronous flashing condition are also presented (Supplementary Section A). The patterns in the background and the background matching stimulus were unique for each presentation. All trials had a single target, but the number of distractors (0, 5, 11, 17 to 23) varied depending on the group size (1, 6, 12, 18 and 24). Because background matching stimulus was patterned, and all colour dynamic stimuli were plain, we also performed another experiment where we compared background matching and colour dynamic stimuli with a unicoloured grey stimulus having the average background colour (see Supplementary Section B).

All participants were undergraduate students from the Indian Institute of Science Education and Research Thiruvananthapuram and were naïve to the experimental hypothesis. Participants had normal or corrected normal vision and gave their written informed consent to participate. The experiments were conducted in accordance with the Declaration of Helsinki (2013, Version 7) and the experiment was approved by Indian Institute of Science Education and Research Thiruvananthapuram Ethics committee. The tracking task involved tracking the movement of a single target circle with a mouse-controlled cursor (represented by a red circle of radius 10 pixels on the screen) for a total period of 5000 ms. The target circle was highlighted (encircled in green) for the initial 1000 ms, and the Euclidean distance between the target and the cursor (tracking error) was recorded thereafter at 16 ms intervals. At the start of each trial, the cursor was positioned at the centre of the screen. Prior to the actual experiments, participants were asked to perform practice trials where black circular objects were presented on a plain white background, with other conditions identical to that in the main experiments. The main experiments consisted of 20 trials per participant, ordered as four blocks based on the stimuli type (i.e., background matching, colour dynamic stimuli at 5, 10 or 15 Hz). The order of presentation of group size within each block and the order of the stimuli type were randomized for every participant.

### Statistics

Data were analyzed in RStudio V 3.3.3 (R Foundation for Statistical Computing, www.R-project.org). A Linear Mixed-Effects Model framework was used due to the repeated measures design of the experiment (*lmer* function from the *lme4* package^58^), with participant ID as the random intercept term. The model fitted the natural log-transformed tracking error as a response variable against group size (i.e., the number of circles presented), stimuli type and the interaction between the two as the fixed effect term. Since it is well-known that the confusion effect saturates after a threshold group size^15,17,27^, the model fitted group size as a quadratic polynomial, which was significantly better than a linear model (χ^2^ = 1030.7, d.f. = 4, *P* < 0.0001), and this was used in the remaining analysis. The main effects of the predictor variables were calculated using the *anova* function^59^, and the P-values from the main model were obtained using the *lmerTest* package^60^.

### Ethic statement

The experiments were conducted in accordance with the Declaration of Helsinki (2013, Version 7) and was approved by Indian Institute of Science Education and Research Thiruvananthapuram Ethics committee. Participants signed an informed consent to participate.

## Electronic supplementary material

Background matching stimulus presentation

Practice stimulus presentation

Colour dynamic stimulus (5 Hz) presentation

Colour dynamic stimulus (10 Hz) presentation

Colour dynamic stimulus(15 Hz) presentation

Supplementary material

Dataset 1

## Data Availability

The data set supporting the results are submitted as supplementary to the article.
