# Supplementary material for: Dynamic colour change and the confusion effect against predation
Source: Sci Rep. 2019 Jan 22;9:274. doi: 10.1038/s41598-018-36541-7 (PMC6342951; doi:10.1038/s41598-018-36541-7)
Supplement: Supplementary file 6 — Supplementary material [file 41598_2018_36541_MOESM6_ESM.docx]

**Supplementary Material:** Dynamic colour change and the confusion effect against predation

Gopal Murali*, Kajal Kumari and Ullasa Kodandaramaiah IISER-TVM Centre for Research and Education in Ecology and Evolution (ICREEE), School of Biology, Indian Institute of Science Education and Research Thiruvananthapuram, Maruthamala PO, Vithura, Thiruvananthapuram, India. 695 551.

**Supplementary material**

**Section A: Tracking error for the synchronously flashing condition**

**General methods**

In this experiment, the tracking task and presentation of the stimulus were identical to that in the main experiment except that all the colour dynamic objects in the group flashed synchronously at the same time. There were a total of 29 participants, and the colour change frequency was set to 15 Hz for the colour dynamic stimulus which were compared with background matching stimulus. The background was same as in the main experiment (see main text).

**Results**

The main effects included in the model - stimuli type (χ^2^=805.14, d.f.= 1, *P*<0.0001) and group size (χ^2^=6074.02, d.f.= 2, *P*<0.0001) - as well as the interaction between the two (χ^2^=271.16, d.f.= 2, *P*<0.0001) were significant. Overall, tracking error increased significantly (t=44.463, *P*<0.0001) with an increase in group size (on average 99.745% difference between 1 and 24). When compared to the background matching stimulus, tracking error was significantly higher for the colour dynamic stimulus (t=28.506, *P*<0.0001). The difference in tracking error between background matching and the colour dynamic stimulus increased significantly with increase in group number (t=15.417, *P*<0.0001).


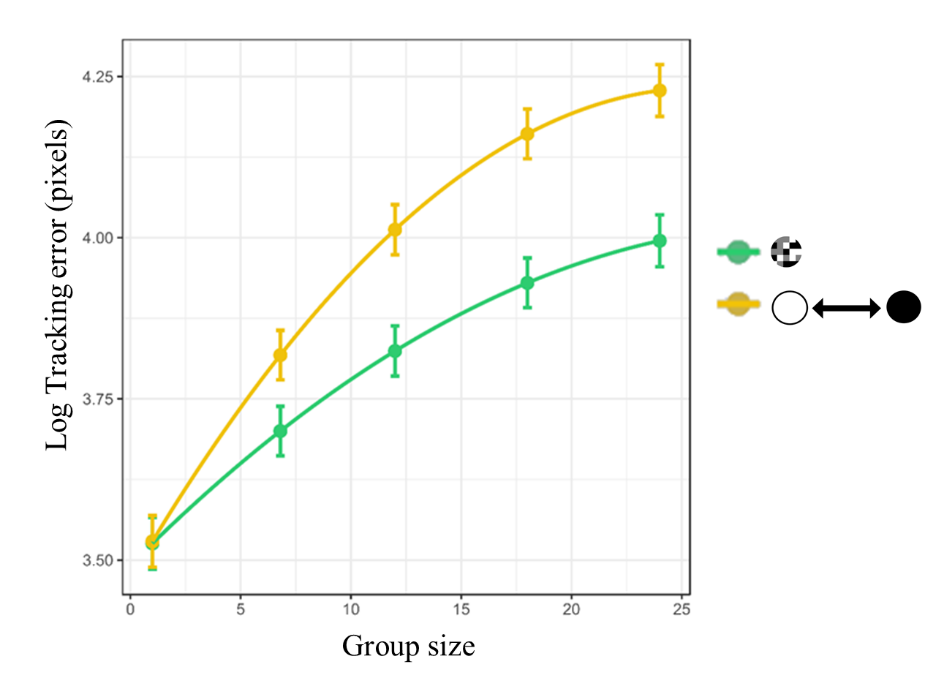


**Figure S1:** Estimated mean and 95% confidence intervals of log transformed tracking error (in pixels) from the Linear Mixed Effects model. green - background matching stimulus; yellow - colour dynamic stimulus with 15 Hz colour change frequency.

**Section B: Tracking error for the asynchronously flashing condition with average grey stimulus**

**General methods**

In this experiment, the tracking task and presentation of the stimulus were identical to that in the main experiment except that the experiment had plain grey stimulus with average colour values of the background (R-128, B-128, G-128). The experiment also had a background matching stimulus and colour dynamic stimulus with colour change frequency of 5 Hz. There were a total of 22 participants in this experiment. The colour dynamic stimulus was allowed to flash asynchronously. The background was same as in the main experiment (see main text).

**Results**

The main effects included in the model - stimuli type (χ^2^=524.912, d.f.=2, *P*<0.0001) and group size (χ^2^=6456.535, d.f.= 2, *P*<0.0001)- as well as the interaction between the two (χ^2^=77.688, d.f.= 4, *P*<0.0001) were significant. Overall, tracking error increased significantly (t=47.769, *P*<0.0001) with an increase in group size (on average 103.34% difference between 1 and 24). When compared to the average grey stimulus, the tracking error was significantly higher for the colour dynamic stimulus (t=14.936, *P*<0.0001) but was lower for the background matching stimulus (t=-7.209, *P*<0.0001). The difference in tracking error between grey and the colour dynamic stimulus increased significantly with increase in group number (t=3.040, *P*=0.0023).


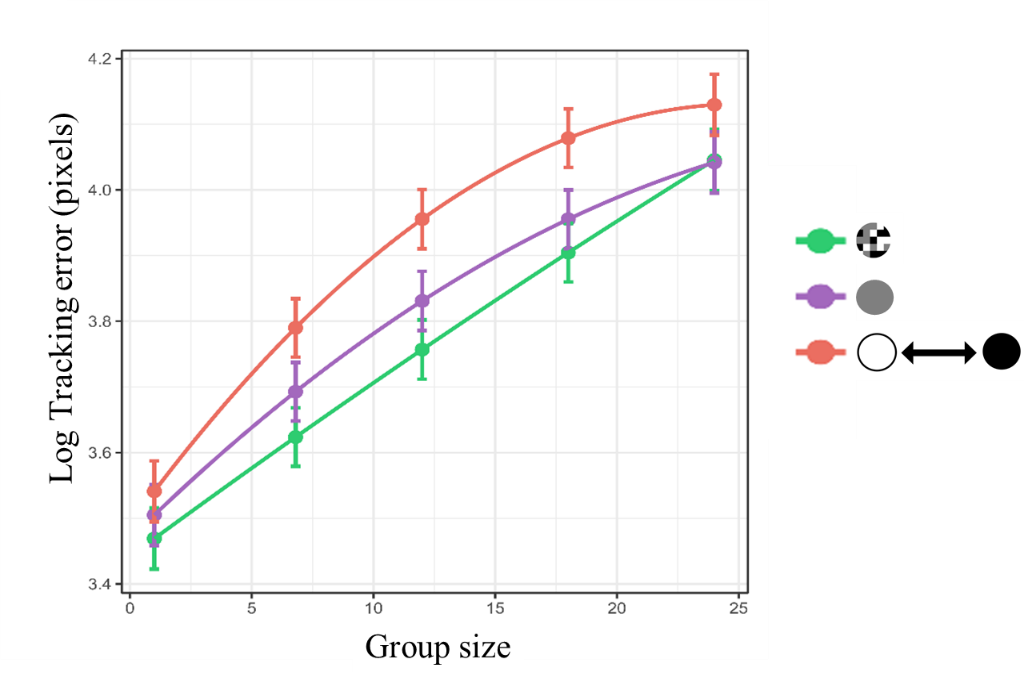


**Figure S2:** Estimated mean and 95% confidence intervals of log transformed tracking error (in pixels) from the Linear Mixed Effects model. green - background matching stimulus; purple - plain grey stimulus; red- colour dynamic stimulus with 5 Hz colour change frequency.

**Section C: Written instructions given to participants**

INSTRUCTIONS

1. Your objective is to keep tracking the movement of a circular target object (encircled in green) with a mouse-controlled on-screen cursor.
2. Move the on-screen cursor (red circle) as close as possible to the circular target object
3. Keep tracking the object with the cursor until the next set of objects appears
4. Position yourself such that the distance between your head and the screen does not change during the experiment
5. Please ensure that you sit directly in front of the screen and not lean towards the monitor screen
6. The experiment will end automatically after 25 seconds
7. Please do not discuss the experiment with others as it may influence the outcome of the experiment
8. Enter your name and click the mouse to start the experiment
